# Pharmacologic Boosting of Atazanavir in Maintenance HIV-1 Therapy: The COREYA Propensity-Score Adjusted Study

**DOI:** 10.1371/journal.pone.0049289

**Published:** 2012-11-09

**Authors:** Laurent Hocqueloux, Philippe Choisy, Gwenaël Le Moal, Françoise Borsa-Lebas, David Plainchamp, Eric Legac, Thierry Prazuck, Xavier de la Tribonnière, Yazdan Yazdanpanah, Jean-Jacques Parienti

**Affiliations:** 1 Service des Maladies Infectieuses et Tropicales, Centre Hospitalier Régional, Orléans, France; 2 Service Universitaire des Maladies Infectieuses et du Voyageur, Hôpital Gustave-Dron, Tourcoing, France; 3 Service des Maladies Infectieuses, Hôpital de la Milétrie, Poitiers, France; 4 Service des Maladies Infectieuses, Hôpital Charles Nicolle, Rouen, France; 5 Laboratoire de Biologie, Centre Hospitalier Régional, Orléans, France; 6 Unité Biostatistique et Recherche Clinique, CHU Côte de Nacre, Caen, France; University of Pittsburgh, United States of America

## Abstract

**Background:**

Among HIV-1 infected patients who achieved virologic suppression, the use of atazanavir without pharmacologic boosting is debated. We evaluated the efficacy and tolerance of maintenance therapy with unboosted atazanavir in clinical practice.

**Methods and Results:**

This multicenter retrospective cohort study evaluated the efficacy of switching HIV-1-infected patients controlled on triple therapy to unboosted (ATV_0_, n = 98) versus ritonavir-boosted atazanavir (ATV/r, n = 254) +2 nucleos(t)ide reverse transcriptase inhibitors. The primary endpoint was time to virologic failure (VF, >200 copies/mL). ATV groups were compared controlling for potential confounding bias by inverse probability weighted Cox analysis and propensity-score matching. Overall and adjusted VF rates were similar for both strategies. Both strategies improved dyslipidemia and creatininemia, with less jaundice in the ATV_0_ group.

**Conclusion:**

In previously well-suppressed patients, within an observational cohort setting, ATV_0_–based triple-therapy appeared as effective as ATV/r- based triple-therapy to maintain virologic suppression, even if co-administered with TDF, but was better tolerated.

## Introduction

Although most patients achieve virologic control with combined antiretroviral therapy (cART), adherence and tolerability issues (particularly metabolic and cardiovascular-associated morbidity) remain important concerns for HIV-infected patients receiving long-term treatment [1], [2]. Unboosted atazanavir (ATV_0_) and tenofovir disoproxil fumarate (TDF) are two antiretrovirals that combine a low pill burden with good tolerability and favorable metabolic profile [3]–[7]. However, a pharmacological interaction occurs during co-administration of ATV and TDF, which has resulted in recommendations to continue low-dose ritonavir (RTV), which might reduce the expected benefit [1], [2], [8]. With this background, not one of the randomized studies demonstrating the non-inferiority of a maintenance strategy with ATV_0_-based triple therapy was conducted in patients receiving TDF [3], [4], [9]. Recently, two cohort studies have demonstrated the durability and safety of maintenance with ATV_0_-based triple therapy co-administered with TDF, but none included direct comparison with a RTV-boosted ATV regimen (ATV/r) [10], [11].

This study compared the efficacy of a switch from ATV/r to ATV_0_, with or without TDF, as a maintenance strategy in pre-treated patients on successful cART.

## Patients and Methods

The COREYA study (COhort with REYAtaz) was a retrospective analysis of a large prospective multicentric cohort of HIV-1-infected subjects in 5 teaching hospitals in France. Patients had virological suppression (plasma HIV RNA <50 cp/mL) while receiving a triple therapy (2 nucleoside reverse transcriptase inhibitors [NRTI] +1 boosted protease inhibitor [PI], 2 NRTI +1 non-nucleoside reverse transcriptase inhibitor [NNRTI] or 3 NRTI), and had switched to ATV-based triple therapy, with or without ritonavir (RTV), between 2004 and 2011. Baseline was defined as the day ATV_0_ or ATV/r was initiated; there was no restriction on the use of TDF.

In this retrospective study, all participants provided informed written consent for the anonymous use of their clinical and biological data for biomedical research at the time their data were entered in the electronic database. The COREYA study was approved by the Institutional Review Board at the Côte de Nacre University Hospital, Caen, France (A10-D07-VOL.10, applicable to all French sites) on June 2010 and waived written informed consent, given the retrospective and observational nature of the current study. In addition, the anonymous use of the electronic database has been approved by the CNIL (Commission National de l’Informatique et des Libertés: http://legimobile.fr/fr/cnil/dec/aut/rech/2011/DR-2011-239/).

The primary endpoint was time to virological failure (single plasma HIV RNA >200 cp/mL; VF>200). Plasma HIV RNA >50 cp/mL (VF>50) was also analyzed. Secondary endpoints were safety (fasting lipids, renal function and hyperbilirubinemia), CD4^+^ reconstitution and T cell residual activation levels, pharmacokinetics, and reasons for change of treatment strategy other than VF.

Data collected at baseline included: demographics, medical and antiretroviral treatment history, genotype (where available for patients with a history of VF), CD4^+^ cell count nadir and plasma HIV RNA viral load (PVL) zenith before treatment, hepatitis B or C co-infection, bilirubinemia and serum creatinine levels, fasting cholesterol and triglycerides.

Follow-up data included: CD4^+^ cell counts, PVL, bilirubinemia and serum creatinine levels, fasting cholesterol, triglycerides, genotype at failure (if any) and change in cART regimen. Blood samples were taken 3 to 4 times a year, as recommended by national guidelines. When available, T cell activation status and plasma ATV trough concentrations (ATV C_trough_) data were collected.

The activation markers (CD38 and HLA-DR) were analyzed on gated CD4^+^ and CD8^+^ T cells and T cell activation levels were mainly defined as the percentages of CD4^+^ and CD8^+^ T cells expressing both CD38 and HLA-DR. T cell activation was measured in freshly collected, EDTA-anticoagulated whole blood and analyzed by flow cytometry (Becton-Dickinson FACS Canto II). Study patients were compared with a group that had undetectable PVL under NNRTI-based triple-therapy and HIV-uninfected healthy donors (personal unpublished data). All treated groups were matched for age, sex ratio, nadir CD4^+^ count, highest PVL before cART and duration with undetectable PVL.

Measurements of ATV C_trough_ were performed using high-performance liquid chromatography. Only samples collected 24±2 hours after the last intake of ATV_0_ or ATV/r were considered.

All genotypes were (re-)interpreted using the ANRS 2011 algorithm (http://www.hivfrenchresistance.org/2011/Algo-2011.pdf).

With a rate for the primary endpoint of 4 per 100 patient-years in the ATV/r group, median accrual and follow-up times of 1 and 2 years, respectively, a one-sided alpha of 0.05, a power of 80%, a true hazard ratio of 1.0 and a proportion of ATV/r (control group) of 66.6%, and a hazard ratio non-inferiority margin of 3.0, the calculated sample size was 241.

Data were expressed as mean ± standard deviation, or median (interquartile range) and percentage. Differences between patients switching to ATV_0_ or ATV/r were compared using Chi-square or Fisher exact tests for categorical variables and two-tailed, unpaired *t* test or Wilcoxon rank sum test for continuous variables.

The association between VF and treatment strategy was evaluated by the hazard ratio and its 95% confidence interval (CI), with a value >1 signifying an increased risk of virologic failure in the ATV_0_ group. Analysis using propensity scores was conducted to limit inclusion biases. Potential indication or ‘channeling’ biases were adjusted for by developing a propensity score for switching to one versus another strategy [12]. Two different marginal structural models were used [13], [14]: inverse probability weighting (IPWT) and matching on the propensity score. The primary endpoint was modeled by IPWT Cox model, after checking the proportionality assumption which was met. Second, a one-to-one greedy 5 to 1 digit technique to match one control (ATV/r group) by one case (ATV_0_ group) nested within the overall cohort was performed. In this matched sample, baseline characteristics included in the propensity score were compared between cases and controls by paired-tests [14]. The probability of endpoint was then modeled in a Cox model with robust sandwich variance estimators including the ATV group as explanatory factor. A *P* value <0.05 was considered significant and all *P* values were two-tailed. Statistical analyses were performed using SAS statistical software, version 9.1 (SAS Institute Inc., Cary, NC).

## Results

### Baseline Characteristics

A total of 352 patients were included, 98 of whom switched to ATV_0_ and 254 to ATV/r. Overall, most patients (191/352, 54%) switched from lopinavir/r-based cART. The main reasons for switching to ATV were to reduce pill burden (218/352, 62%) and side effects (91/352, 26%). Sixty-one patients (17%) had a history of VF; genotyping was available in 65%, and resistance-encoding mutations for ≥1 drug in the NRTI, NNRTI and PI classes were found in 53%, 53% and 24% of the available sequences, respectively. There were significant differences between the two groups (boosted and unboosted) at baseline (Table 1). Factors significantly associated with a switch to ATV without ritonavir rather than a switch to ATV with ritonavir were female sex, asymptomatic HIV infection (according to the CDC classification), higher CD4 cells nadir, high number of previous lines of treatment, prior treatment with 3 NRTI, longer time with undetectable plasma viral load, higher CD4 cell count and more dyslipidemia as the reason for the switch. Propensity score matching limited these differences (as shown in Table 1). The mean follow-up was 2.2±1.7 years, which was similar in the two groups.

**Table 1:** Baseline characteristics and main outcomes for patients switching to unboosted (ATV_0_) versus boosted (ATV/r) atazanavir-based regimens in the overall population and in the propensity-matched subgroups.

	Variables	overall cohort			matched cohort		
		unboosted(n = 98)	boosted(n = 254)	*P*	unboosted (n = 72)	boosted (n = 72)	*P*
*At baseline*	age, years, mean (SD)	42.8 (11.2)	43.4 (10.9)	0.69	42.2 (11.2)	43.4 (10.1)	0.65
	male gender, n (%)	54 (55)	169 (67)	0.049	39 (54)	41 (57)	0.86
	BMI, mean (SD)	24.0 (3.9)	23.5 (4.0)	0.31	24.4 (4.0)	24.0 (4.5)	0.53
	Co-infection, n (%)						0.71
	HBV	7 (7)	5 (2)	0.05	3 (4)	3 (4)	
	HCV	10 (10)	30 (12)		6 (8)	9 (12)	
	CDC classification, n (%)			0.005			
	A	63 (64)	141 (56)		50 (69)	47 (65)	0.83
	B	25 (26)	47 (18)		15 (21)	18 (25)	
	C	10 (10)	66 (26)		7 (10)	7 (10)	
	Lowest CD4, cells/µL, mean (SD)			0.008			0.73
	>500	8 (8)	3 (1)		5 (7)	3 (4)	
	200–500	50 (51)	113 (45)		39 (54)	42 (58)	
	<200	40 (41)	138 (54)		28 (39)	27 (38)	
	Highest PVL, Log copies/mL, mean (SD)	5.0 (0.7)	5.1 (0.8)	0.48	5.0 (0.7)	5.1 (0.7)	0.65
	Prior lines of treatment, mean (SD)	3.7 (2.7)	3.0 (2.8)	0.006	3.3 (2.5)	3.6 (2.9)	0.82
	History of virological failure, n (%)	23 (23)	38 (15)	0.08	17 (24)	16 (22)	>0.99
	Last regimen before switch, n (%)			0.009			0.76
	2 NRTI +1 PI/r	80 (92)	225 (89)		61 (85)	64 (89)	
	2 NRTI +1 NNRTI	11 (11)	26 (10)		8 (11)	6 (8)	
	3 NRTI	7 (7)	3 (1)		3 (4)	2 (3)	
	Years with PVL <50 cp/mL, mean (SD)	2.9 (3.3)	1.0 (1.5)	<0.0001	1.8 (2.1)	1.8 (2.4)	0.63
	Last CD4, cells/µL, mean (SD)	616 (274)	494 (241)	<0.0001	598 (258)	621 (270)	0.61
	Reason for switch, n (%)			<0.0001			0.13
	Simplification	47 (48)	171 (67)		35 (49)	43 (60)	
	Dyslipidemia	22 (22)	12 (5)		16 (22)	6 (8)	
	Tolerability	27 (28)	64 (25)		20 (28)	21 (29)	
	Other	2 (2)	7 (3)		1 (1)	2 (3)	
	Backbone associated with ATV, n (%)			<0.0001			0.04
	contains ABC	29 (30)	141 (48)		24 (33)	33 (46)	
	containsTDF	60 (61)	88 (34)		42 (58)	26 (36)	
	contains ABC and TDF	5 (5)	9 (4)		4 (6)	6 (8)	
	contains neither ABC nor TDF	4 (4)	36 (14)		2 (3)	7 (10)	
*At LOCF*	Duration of follow-up, years, mean (SD)	2.2 (1,6)	2.1 (1.7)	0.64	2.3 (1.7)	2.2 (1.7)	0.6
	Viral failure>200 cp/mL, n (%)	3 (3.1)	13 (5.1)	0.39	3 (4.1)	3 (4.1)	0.94
	Discontinuation without VF, n (%)	17 (17)	96 (38)	0.0008	9 (13)	33 (46)	0.0001

Abbreviations: SD, Standard Deviation; BMI, Body Mass Index; HBV, Hepatitis B Virus; HCV, Hepatitis C virus; PVL, Plasma Viral Load; NRTI, Nucleos(t)ide Reverse Transcriptase Inhibitor; PI, Protease Inhibitor; NNRTI, Non Nucleoside Reverse Transcriptase Inhibitor; ATV, Atazanavir; ABC, Abacavir; TDF, Tenofovir. LOCF, Last Observation Carried Forward.

### Primary and Secondary Virologic Endpoints

**Figure 1:**
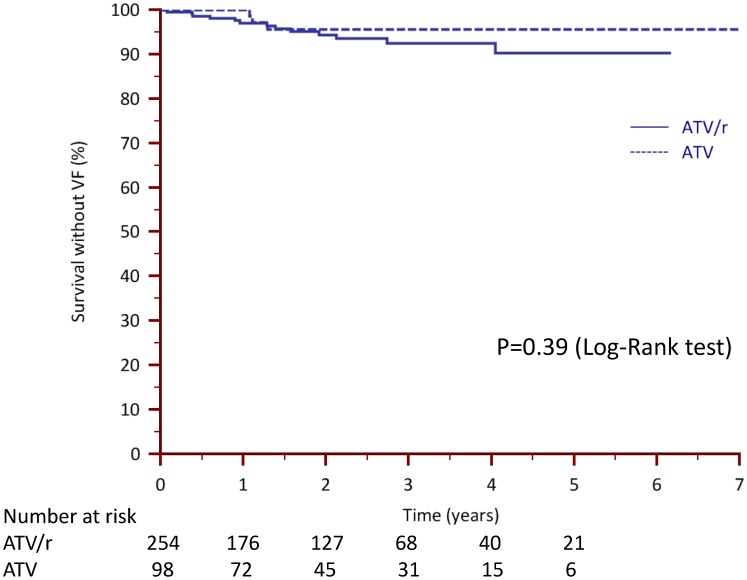
Kaplan-Meier curves of time without viral failure (VF, >200 cp/mL) in patients switching to to unboosted (ATV_0_) versus boosted (ATV/r) atazanavir-based regimens in the overall population.

Overall, VF>200 occurred in 3/98 (3.1%) and 13/254 (5.1%) patients in the ATV_0_ and ATV/r groups, respectively (p = 0.39) (Figure 1). In the IPWT-analysis, risk of VF>200 was not statistically different (HR 0.85, 95%CI [0.38–1.92], p = 0.70); the upper boundary of the 95% CI was below the pre-defined non-inferiority margin for the HR. Corresponding values for VF>50 were 3/98 (3.1%) and 27/254 (10.6%; p = 0.025), which translated to a significant difference favouring ATV_0_ over ATV/r in the IPWT-analysis (HR 0.39, 95%CI [0.19–0.80], p = 0.011).

**Figure 2:**
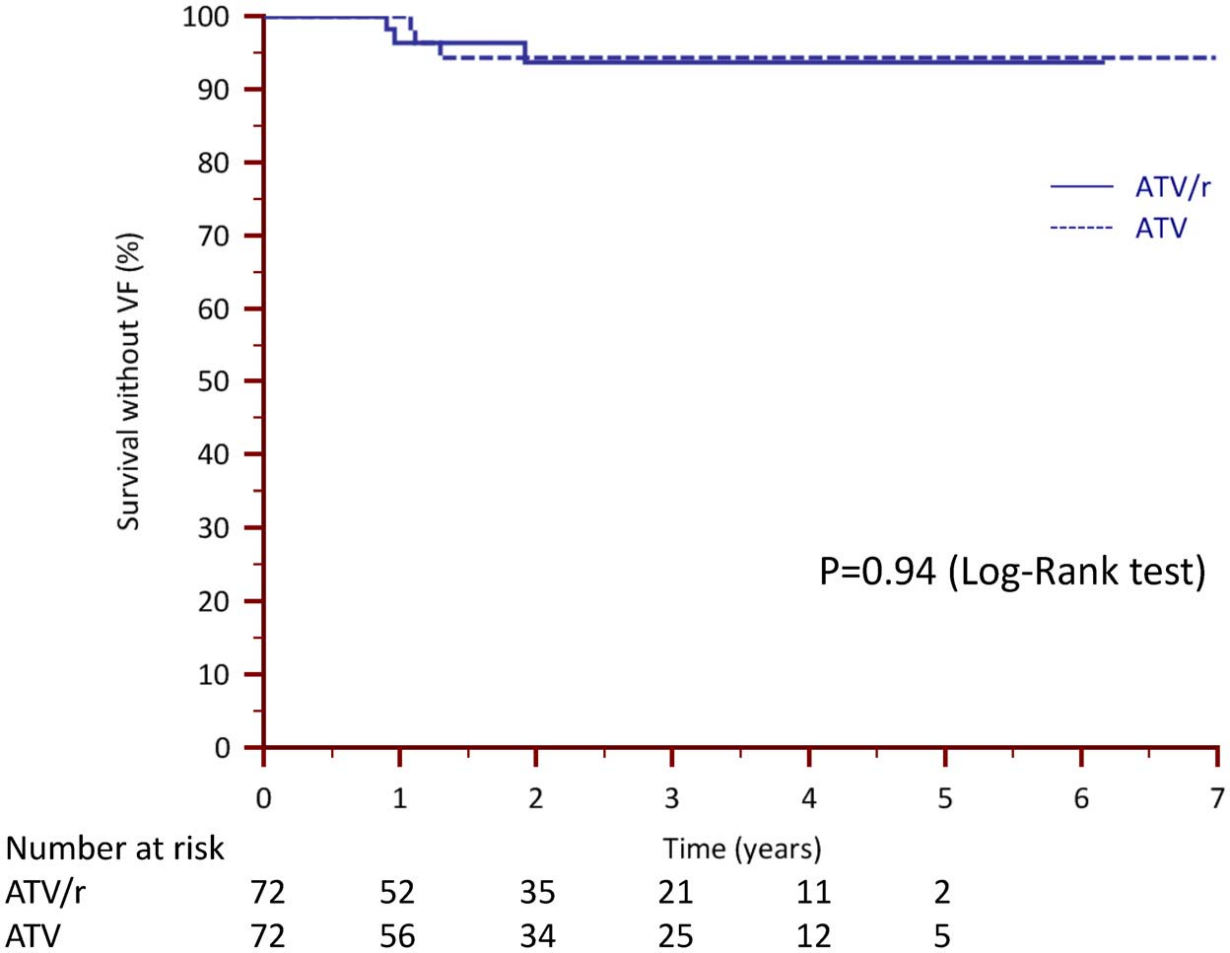
Kaplan-Meier curves of time without viral failure (VF, >200 cp/mL) in patients switching to to unboosted (ATV_0_) versus boosted (ATV/r) atazanavir-based regimens in propensity-matched subgroups.

For analyses in the propensity-matched subgroups, 3/72 (4.1%) and 3/72 (4.1%) patients experienced VF>200 (HR 0.95, 95%CI [0.25–3.61], p = 0.94) in the ATV_0_ and ATV/r groups, respectively (Figure 2); results for the VF>50 analysis were similar. Overall, and in the ATV_0_ group, there was a trend for less VF in patients receiving TDF.

### Other Secondary Endpoints

During follow-up, CD4^+^ count increased from baseline (p<0.0001), with both ATV_0_ and ATV/r groups showing similar immune reconstitution (p = 0.68). There was no difference between the ATV_0_, ATV/r and NNRTI groups in terms of residual T cell activation levels. HIV-uninfected donors (n = 21) had much lower T cells activation levels than all three treated groups (p = 0.002 and p = 0.02, respectively).

In the ATV_0_ group, the mean C_trough_ (n = 66) was 200±176 ng/mL (36% of values were >200 ng/mL, that is the recommended threshold to be above). Of note, there was no difference in ATV_0_ C_trough_ between patients with or without TDF in the backbone (200±188 vs 205±153, respectively, p = 0.64). There was no association between ATV_0_ C_trough_ and VF (216 if VF, 199 if no VF, p = 0.43). Patients with ATV_0_ C_trough_ <200 ng/mL (n = 42) had similar VF>200 rate than patients with ATV_0_ C_trough_ >200 ng/mL (3% vs 12%, p = 0.21, Log-Rank test). In the ATV/r group C_trough_ (n = 30) values were much higher (949±1401, p<0.0001) than in the ATV_0_ group.

LDL-cholesterol (n = 153, −0.29±0.95 mmol/L, p<0.001) and triglycerides (n = 173, −0.54±1.26, p<0.001), but not HDL cholesterol (n = 166, 0.01±0.34, p = 0.79), decreased from baseline in both groups, with no statistically significant difference between groups. There was also a significant decrease in creatinemia (n = 337, −3.8±15 µmol/L, p = 0.001). No significant difference was found between the patients according to the ATV group, or the presence or absence of TDF. Hyperbilirubinemia grade 3–4 was less frequent in ATV_0_ than in ATV/r group (9% vs 30%, p<0.0001).

Overall, discontinuation for other reasons than VF occurred in 113/352 patients (32%), after a mean 1.5 years. Main causes were: clinical tolerability issues and patients’ request (n = 78), physician’s decision to change strategy (n = 9), pregnancy (n = 8), dyslipidemia (n = 3), regimen simplification (n = 3), death (n = 1) and miscellaneous (n = 11). The discontinuation rate differed markedly between groups, with a much lower rate for ATV_0_ versus ATV/r, even after adjustment for propensity score (Table 1).

## Discussion

This study suggests that, in previously well-controlled patients, ATV_0_ is an effective alternative to ATV/r for maintaining long-term viral suppression, even in treatment-experienced patients and in the presence of TDF. In this setting, ATV_0_ is better tolerated than ATV/r.

For the main analysis, we chose to define VF as >200 cp/mL rather than >50 cp/mL because the lower threshold may categorize an unacceptably high number of patients who ultimately re-suppress to <50 cp/mL without a change in ART as having VF [15], [16]. The results show that the failure rate at 1, 2 and 3 years using the 200 cp/mL threshold is remarkably low in patients with ATV_0_ (0, 4 and 4% respectively) and similar to published figures [3], [4], [11]. In one study, using a VF threshold of <400 cp/mL, the failure rate was <2%, <3% and <5% at 1, 2 and 3 years, respectively [11]. In this group, the only risk factor for failure was co-infection with hepatitis C, and the authors suggested a link between substance abuse and poor compliance [11]. In our study, the rate of co-infection was low and patients had good control prior to the switch to ATV_0_ (almost 3 years). In addition, the reason patients in our study switched to ATV_0_ (to reduce side effects or lower pill burden) is likely to perpetuate a high adherence rate.

It is noteworthy that our patients frequently had ATV_0_ C_trough_ below the recommended threshold without an increased risk of VF. Poor adherence to ATV_0_ is very unlikely in the context of patients with long-term viral suppression, good immunologic reconstitution and low immune activation. This suggests that, in patients with long-term viremia suppression, the current recommended cut-off of >200 ng/mL may be overestimated.

Overall, TDF is associated with trend toward a better virological control. In the context of co-administration with ATV_0_, which does not allow complete coverage of the day in more than half of patients, the long half-life of TDF may offer an advantage over abacavir. Moreover, in our study, TDF did not have a negative impact on the pharmacokinetics of ATV_0_, as there was no more under-dosing than in the group without TDF. Our results are similar to those reported by Calcagno *et al* but contradict previously reported pharmacokinetic studies [8], [17]. In highly pre-selected patients within a cohort study, we did not observe this well described pharmacokinetic interaction [8], however our study was not designed to assess such observations. Therefore, this result should be interpreted with caution and therapeutic drug monitoring may be warranted on a case-by-case basis.

ATV is associated with a good metabolic profile and low levels of insulin-resistance. Indeed, a recent study reported that ATV, even boosted, is not associated with an excess risk for coronary artery disease or stroke [18]. The use of unboosted ATV avoids ritonavir-associated toxicity and also reduces ATV systemic, which has been shown to decrease the risk of dyslipidemia, renal impairment and jaundice [3], [4], [11], [19] and also perhaps nephrolithiasis [11]. It is interesting to note in our study that the risk of ATV_0_ discontinuation for causes other than VF was significantly lower than in the ATV/r group. These findings suggest that ATV_0_ is better tolerated than ATV/r. The reduction of side effects is likely to be one of the main causes of the durability of ATV_0_-based regimen, as it has been demonstrated that side effects are strongly associated with decreased average adherence [20], leading to VF [21].

The main limitation of this study is its retrospective design, which can introduce important biases, particularly channelling bias. For example, the use of abacavir has been associated with an increased risk of myocardial infarction in cohort studies despite multivariable adjustments [22], [23] but not in a carefully matched case-control study [24] or in randomized controlled trials [25]. In our study, the ATV/r boosted group was clearly disadvantaged at baseline, combining markers of poor prognosis (more previous AIDS-defining illnesses, lower CD4^+^ counts, etc). This might explain the better secondary virologic outcome (VF<50) observed in the ATV_0_-based group despite the use of the sophisticated IPWT adjustment. We attempted to overcome this by incorporation of an analysis based on propensity score matching subgroup nested in our cohort and therefore excluding patients too different to be matched. The latter analysis did not confirm the better virologic outcome (VF<50) in the ATV_0_ group. More importantly, our results appear to be consistent with current literature, including from randomized controlled trials [3], [9].

In conclusion, maintenance of patients on successful cART for ≥1 year with ATV_0_-based triple therapy is associated with low rates of VF and discontinuation without VF compared with ATV/r. In this setting, the use of TDF had no deleterious impact on ATV_0_ C_trough_ and virologic efficacy.
